# Data Resource Profile: The Virtual Cardio-Oncology Research Initiative (VICORI) linking national English cancer registration and cardiovascular audits

**DOI:** 10.1093/ije/dyab082

**Published:** 2021-06-05

**Authors:** Michael J Sweeting, Clare Oliver-Williams, Lucy Teece, Catherine A Welch, Mark A de Belder, Briana Coles, Paul C Lambert, Lizz Paley, Mark J Rutherford, Lucy Elliss-Brookes, John Deanfield, Mick D Peake, David Adlam, the VICORI Collaborative

**Affiliations:** 1 Biostatistics Research Group, Department of Health Sciences, University of Leicester, Leicester, UK; 2 National Cancer Registration and Analysis Service, Public Health England, London, UK; 3 National Institute for Cardiovascular Outcomes Research, Barts Health NHS Trust, London, UK; 4 Department of Medical Epidemiology and Biostatistics, Karolinska Institutet, Stockholm, Sweden; 5 Institute of Cardiovascular Science, University College London, London, UK; 6 Department of Respiratory Medicine, University of Leicester, Leicester, UK; 7 Department of Cardiovascular Sciences and NIHR Leicester Biomedical Research Centre, University of Leicester, Leicester, UK

**Keywords:** Data resource, cardiovascular, oncology, cardio-oncology

Key Features

- The Virtual Cardio-Oncology Research Initiative (VICORI) programme brings together English national cancer data and six national cardiovascular disease audits to investigate the interplay between cardiovascular disease and cancer.
- The VICORI data resource captures adults (aged 18+ years) who were hospitalized for cardiac disease, had a cardiac procedure and/or a cancer diagnosis alongside information on their treatment and outcomes. These data are routinely collected and submitted to health care registries and are linked using a unique health service number.
- Detailed data on cancer and cardiac diagnosis, treatment, outcomes, previous and subsequent hospital diagnoses and operations, and mortality are available from 6.2 million cancer diagnoses between 1995 and 2018, and 3.8 million cardiac hospital admissions/procedures between 1999 and 2018.
- The VICORI cohort will be updated on a rolling basis with annual updates from the audits.
- Interested research collaborators can contact VICORI researchers at [vicori@le.ac.uk].

## Data resource basics

### Background

Cancer and cardiovascular disease (CVD) are the most common causes of morbidity and mortality worldwide. Improvements in treatment strategies for both CVD and cancer have resulted in significant improvements in survival and, as a result, there is an increasing population of patients who now live with both conditions.^1–3^ It is well known that cancer and its treatment increase the risk of CVD.^4–6^ Yet a detailed understanding of the underlying relationship between these two conditions and their respective treatments, including both positive and negative modulation of risk, is lacking. This is partly because few cohorts have been large enough to conduct detailed investigations. To address this, the Virtual Cardio-Oncology Research Initiative (VICORI) has linked national cardiac and cancer registries to create a resource of a larger scale and with longer follow-up than typical investigator-led studies.

### Linking the English national cancer registry with cardiac disease audits

VICORI is an initiative to link data from the English National Cancer Registration and Analysis Service (NCRAS), part of Public Health England (PHE),^7^ with national cardiac audits held by the National Institute for Cardiovascular Outcome Research (NICOR).^8^^,^^9^ The goals are to: (i) provide a quality-assured data resource for research into cancer and cardiac disease; and (ii) identify new scientific avenues that will further knowledge of cardio-oncology through a portfolio of research projects aligned with the VICORI programme grant. These research projects will study how existing conditions and related treatments affect subsequent disease risk and will optimize patient management through informing evidence-based guidelines.

**Figure 1:**
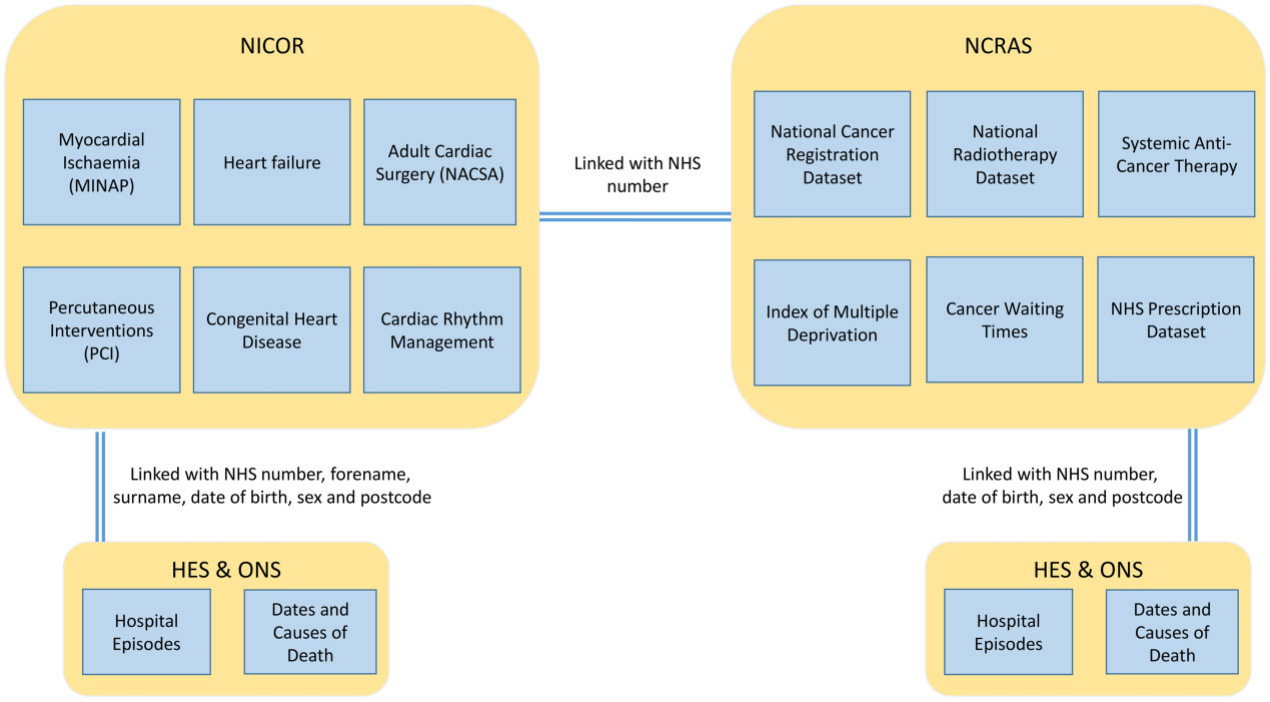
Linkages available between electronic health records within the Virtual Cardio-Oncology Research Initiative (VICORI) resource. HES, Hospital Episode Statistics; MINAP, Myocardial Ischaemia National Audit Project; NACSA, National Adult Cardiac Surgery Audit; NCRAS, National Cancer Registration and Analysis Service; NHS, National Health Service; NICOR, National Institute for Cardiovascular Outcome Research; ONS, Office for National Statistics; PCI, Percutaneous Coronary Interventions.

The VICORI programme has created the world’s first whole-country cardio-oncology research platform from multisource electronic health records (EHR) through the linkage of NCRAS with six cardiac audits held by NICOR,^8^ Hospital Episode Statistics (HES)^10^ and mortality data from the Office for National Statistics (ONS).^11^ The linkages are detailed in Figure 1. England is not the only country with national cancer and CVD registries; Sweden is the only other country with a national myocardial infarction (MI) registry, and also has a national cancer registry,^12^^,^^13^ although other countries have more specific registries (e.g. Belgium’s ST-elevation Registry) and non-disease specific national hospital datasets (e.g. Denmark’s National Patient Registry). However, the English cardiac audits are considerably larger than the Swedish heart disease registry, which began in 2009 and has approximately 80 000 new cases a year. VICORI has records for over 3.8 million cardiac events since 1999, with over 180 000 cases a year since 2010. Similarly, there are markedly more cancer cases recorded in English cancer registries (7.9 million between 1999 and 2017) than in the Swedish cancer registry (2.2 million between 1999 and 2017).^14^

### The National Cancer Registration and Analysis Service

NCRAS is the population-based cancer registry for England^7^ (Table 1). It collects, quality-assures and analyses data on all people in England diagnosed with malignant and some pre-malignant neoplasms. National coverage began in 1971 with data originally recorded on eight regional databases. There are approximately 300 000 malignant tumours diagnosed in England each year, for which NCRAS processes around 25 million records (i.e. multiple records for each tumour). Standardized coding from 2013 onwards follows the International Classification of Diseases for Oncology (ICD-O3) classification; before this ICD-O2 was used.

**Table 1:** Summary of key data items collected by the National Cancer Registration and Analysis Service (NCRAS), National Institute for Cardiovascular Outcomes Research (NICOR) audits, and electronic health record sources

Source	Description	Coding	Fields	Years data available
**National Cancer Registration and Analysis Service (NCRAS) Audits**				
NCRAS Disease Registry: Cancer Registration	National registry: patients diagnosed with cancer (ICD-10 C00-97, D00-48)	Manual extraction from pathology records by trained cancer registration officers	Patient level (date of birth, sex, ethnicity, postcode at diagnosis, comorbidity score, performance status, deprivation) Tumour level (site of neoplasm, morphology, grade, stage, pathology, site-specific fields) Diagnosis level (date of incidence, basis of diagnosis, route to diagnosis) Treatment level [date of treatment event, type (surgery/radiotherapy/chemotherapy)] Mortality information: date of death, full coded causes of death, location of death (from linked ONS data) Hospital admissions, diagnoses and procedures (from linked HES data)	1995^a^–present
NCRAS Disease Registry: National Radiotherapy Dataset (RTDS)	Detailed information from all NHS Acute Trust providers of radiotherapy services in England	Radiotherapy providers submit the data and PHE validates and quality assures the data	Decision to treat, radiotherapy priority and intent, treatment start date, radiotherapy prescription, exposures, OPCS primary procedure code	April 2009-present
NCRAS Disease Registry: Systemic Anti-Cancer Therapy (SACT)	Detailed information from all organizations providing systemic anti-cancer treatment therapies in, or funded by, the NHS in England	Data are entered during the course of care by clinicians, nurses, pharmacists and other health care providers	Demographic and consultant details, clinical status, programme and regimen, cycle, drug details, outcome	April 2012-present
**National Institute for Cardiovascular Outcomes Research (NICOR) Audits**				
NICOR Disease Audit: Myocardial Ischaemia National Audit Project (MINAP)	National audit: patients admitted to hospital with acute coronary syndromes	Data abstracted from clinical records are directly submitted via the secure web portal, or by exporting data from hospital clinical information systems, by clinicians or trained clinical audit staff	>120 fields including phenotype (STEMI, NSTEMI, unstable angina), CVD history, ejection fraction, risk factors, cardiac markers, treatment at hospital, prescription of preventive medications on discharge, also includes specific fields for discharge diagnosis of Takotsubo Cardiomyopathy	October 2000-March 2019
NICOR Disease Audit: The National Heart Failure Audit	National audit: patients with an unscheduled admission to hospital who are discharged with a primary diagnosis of heart failure	Data abstracted from clinical records are directly submitted via the secure web portal, or by exporting data from hospital clinical information systems, by clinicians or trained clinical audit staff	>140 fields including CVD medical history, risk factors, cardiac markers, HF diagnosis, medications prior to admission and on discharge	September 2006-March 2019
NICOR Procedure Audit: The National Adult Cardiac Surgery Audit	National audit: collects data on all major heart operations carried out on NHS patients in the UK. Voluntary data also collected from a number of Irish and UK private surgical units	Data abstracted from clinical records are directly submitted via the secure web portal, or by exporting data from hospital clinical information systems, by clinicians or trained clinical audit staff	>190 fields including procedures (aortic surgery, CABG, valve replacement/repair) and devices, CVD history, ejection fraction, risk factors, postoperative and discharge information	December 2002-March 2019
NICOR Procedure Audit: The National Adult Percutaneous Coronary Interventions	National audit: collects data on all PCI (angioplasty) techniques carried out on NHS patients in the UK	Data abstracted from clinical records are directly submitted via the secure web portal, or by exporting data from hospital clinical information systems, by clinicians or trained clinical audit staff	>120 fields including medical history, cardiac anatomy, PCI procedure, cardiac markers, in-hospital outcomes	June 1999-March 2019
NICOR Procedure Audit: The National Congenital Heart Disease Audit	National audit: infants, children, adolescents and adults undergoing interventions for paediatric and congenital heart disease	Data abstracted from clinical records are directly submitted via the secure web portal, or by exporting data from hospital clinical information systems, by clinicians or trained clinical audit staff	>60 fields including procedures (bypass, catheter, diagnostic), antenatal diagnosis, risk factors, in-hospital outcomes (deaths and complications)	1997–2019 (data due to be included)
NICOR Procedure Audit: The National Audit of Cardiac Rhythm Management (CRM)	National audit: implanted cardiac devices and all patients receiving interventional procedures for management of cardiac rhythm disorders	Data abstracted from clinical records are directly submitted via the secure web portal, or by exporting data from hospital clinical information systems, by clinicians or trained clinical audit staff	ECG indications, ejection fraction, procedures (e.g. new pacemaker, generator change), complications, CVD history, ablation procedures, medications, in-hospital and follow-up information, QoL/PROMs indicators	January 2006–March 2019
**Electronic Health Record sources**				
Secondary care: Hospital Episode Statistics (HES)	National data warehouse of secondary care admissions, including inpatient, outpatient and accident and emergency admissions	Trained non-clinical coders record data based on the discharge summary, weeks after discharge	Up to 20 ICD-10 diagnosis codes, and all procedures and interventions recorded through OPCS-4, geographical information	2000–present
Mortality data: Office for National Statistics (ONS)	National census of all deaths and causes of death taken from the death certificate	Doctor completes death certificate with cause of death; trained non-clinical coders add ICD codes	Date of death, primary underlying cause of death and up to 14 secondary causes, recorded using ICD-10, place of death	1990–present

a Historical cancer registrations before 1995 are available but data completeness and case ascertainment is less reliable.

CABG, coronary artery bypass graft; CVD, cardiovascular disease; HF, heart failure; NCRAS, National Cancer Registration and Analysis Service; NICOR, National Institute for Cardiovascular Outcomes Research; NSTEMI, non-ST-elevation myocardial infarction; PCI, percutaneous coronary intervention; PHE, Public Health England; PROMs, patient reported outcome measures; STEMI, ST-elevation myocardial infarction; TC, Takotsubo cardiomyopathy; QoL, quality of life; ICD, International Classification of Diseases; ONS, Office for National Statistics; HES, Hospital Episode Statistics; OPCS, Office of Population Censuses and Surveys; NHS, National Health Service.

### The National Institute for Cardiovascular Outcome Research

NICOR collects national audit data and produces analyses to enable hospitals and health care improvement bodies to monitor and improve the quality of care and outcomes of cardiac disease patients^8^^,^^9^ and manages the National Cardiac Audit Programme (NCAP). NICOR currently receives data on over 300 000 records per year across six clinical areas: two concerned with particular disease processes (heart attacks and heart failure), and four that cover delivery of specific services (procedures for patients with congenital heart disease, percutaneous coronary intervention (PCI), cardiac surgery and management of cardiac rhythm abnormalities) (Table 1).

### Exemplar patient pathway

**Figure 2:**
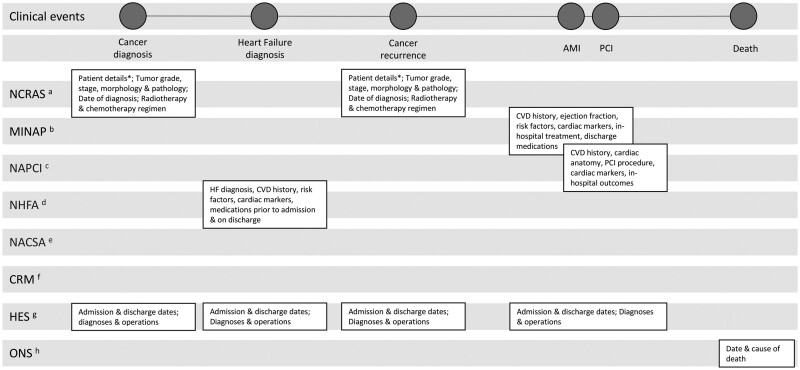
A hypothetical patient’s health care journey demonstrating data available in the datasets included in the Virtual Cardio-Oncology Research Initiative (VICORI) resource. *date of birth, sex, ethnicity, postcode at diagnosis, comorbidity score, performance status, deprivation; ^a^NCRAS, National Cancer Registration and Analysis Service; ^b^MINAP, Myocardial Ischaemia National Audit Project; ^c^NAPCI, National Audit of Percutaneous Coronary Interventions; ^d^NHFA, National Heart Failure Audit; ^e^NACSA, National Adult Cardiac Surgery Audit; ^f^CRM, National Audit of Cardiac Rhythm Management; ^g^HES, Hospital Episode Statistics; ^h^ONS, Office for National Statistics; AMI, acute myocardial infarction; CVD, cardiovascular disease; HF, heart failure; PCI, percutaneous coronary interventions.

Figure 2 illustrates how the different sources within VICORI capture different aspects of a hypothetical patient’s journey. Linkage between NCRAS and the six national cardiac audits allows detailed diagnostic, treatment and service provision information, not available in routine secondary care sources such as HES, to be exploited for research purposes. Through this, the VICORI programme will further knowledge into biological mechanisms, treatment effects and the effectiveness of health care delivery for both cancer and cardiac disease patients.^15^

### Funding sources, organization and key principles

VICORI is a 5-year (2017–22) programme of research jointly funded by Cancer Research UK and the British Heart Foundation. VICORI is led from the University of Leicester by co-principal investigators D.A. and M.P., with research support from the Biostatistics Research Group at the University of Leicester. Along with institutional partners at PHE and NICOR, the programme steering committee consists of academic collaborators from the University of Leicester, University of Oxford, University of Birmingham, University College London, University of Leeds, Imperial College London and the Royal Marsden NHS Trust.

VICORI is built on four key principles; (i) collaboration: working with external researchers to facilitate access to the data resource (see Data resource access); (ii) legacy: ongoing commitment to the VICORI data resource beyond the 5-year programme duration; (iii) dissemination: ensuring maximal coverage and understanding of study findings; and (iv) patient and public involvement: centring patients to ensure research strategy and governance is carried out with, rather than for, them.

### Ethical clearance

The research programme has received favourable ethical opinion from the North East—Newcastle & North Tyneside 2 Research Ethics Committee (REC reference 18/NE/0123).

## Data collected

### The National Cancer Registration and Analysis Service

NCRAS collects and curates the national cancer registration dataset for England, including details on the patient, cancer type (including morphology), how advanced the cancer is and treatments. Identification of cases (‘case ascertainment’) is very high.^16^^,^^17^ Data completeness has improved in recent years; for example stage at diagnosis and ethnicity are now better recorded.^7^ However completeness is variable for other data items, such as performance status, due to limitations in data collection by health care providers. Treatment information is available from data directly processed by NCRAS as well as from linkage to e-prescribing systems for chemotherapy [the Systemic Anti-Cancer Therapy Dataset (SACT)],^18^ radiotherapy machines [the National Radiotherapy Dataset (RTDS)]^19^ and activity data from HES (for inpatient, outpatient and accident and emergency admissions).^10^ Linkage to HES provides further in-hospital information before and after diagnosis, including diagnosis and procedure codes, as well as the opportunity to improve completeness of some variables and check data quality. Linkage to ONS provides data on date and cause of death. Table 1 provides a summary of data items available from NCRAS.

### The National Institute for Cardiovascular Outcome Research

The six national cardiovascular audits administered by NICOR are: (i) the Myocardial Ischaemia National Audit Project (MINAP)^20^; (ii) the National Heart Failure Audit (NHFA); (iii) the National Adult Cardiac Surgery Audit (NACSA); (iv) the National Audit of Percutaneous Coronary Interventions (NAPCI); (v) the National Congenital Heart Disease Audit (NCHDA); and (vi) the National Audit of Cardiac Rhythm Management (NACRM) including devices and ablation datasets (Table 1). The audits are completed by participating hospitals in England [all National Health Service (NHS) hospitals and some private hospitals] and, in some audits, other countries in the UK. The VICORI partnership has gained permission to obtain data from each of the NICOR audits for patients in England.

A strength of the combined NICOR datasets is detailed recording of in-hospital cardiac care and procedures. Available data include but are not limited to: phenotypic specification of acute coronary syndrome admissions (ST-elevated MI, non-ST-elevated MI, unstable angina, cardiomyopathy), severity (ejection fraction, number of vessels, cardiogenic shock), pre-existing comorbidities, detailed in-hospital treatment information, discharge medications, procedures, care pathways and outcomes. Case ascertainment of myocardial infarctions (MI) in MINAP is not complete, but it is improved by linkage with HES and ONS.^21^

### Hospital Episode Statistics

HES is a nationwide dataset of all admissions to NHS hospitals in England. International Statistical Classification of Diseases and Health-Related Problems 10th revision (ICD-10) codes record medical diagnoses, and the Office of Population Censuses and Surveys Classification of Interventions and Procedures (OPCS) Version 4 is used to record the procedures that are undertaken (Table 1).

### Office for National Statistics mortality data

Dates of death for deceased patients in the VICORI cohort are supplied by ONS. Causes of death, extracted from death certificates using ICD-10 codes, are available for all cancer patients, and will become available for patients in NICOR audits at the next periodic update (Table 1).

### Data size

**Figure 3:**
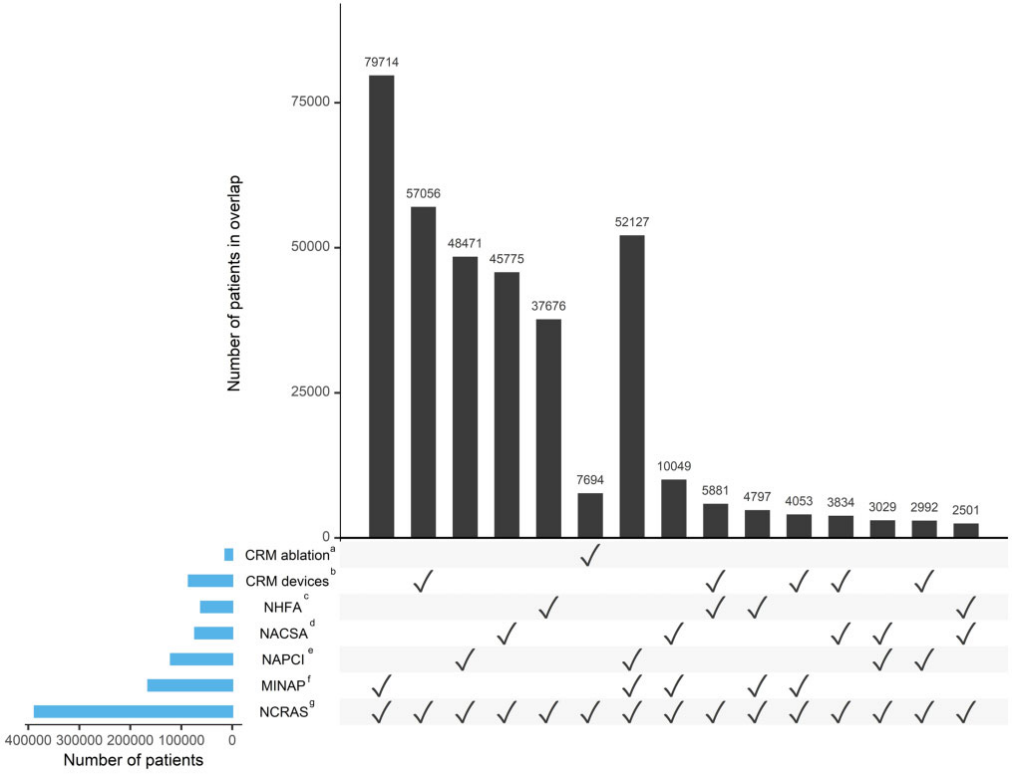
Number of patients in the National Cancer Registration and Analysis Service (NCRAS) (1995–2018) who also feature in one or more of the National Institute for Cardiovascular Outcome Research (NICOR) audits. Plot is restricted to the top 15 intersections, ordered by degree and then frequency. ^a^CRM ablation, Cardiac Rhythm Management ablation interventional procedures audit; ^b^CRM devices, Cardiac Rhythm Management devices audit; ^c^NHFA, National Heart Failure Audit; ^d^ NACSA, National Adult Cardiac Surgery Audit; ^e^NAPCI, National Audit of Percutaneous Coronary Interventions; ^f^MINAP, Myocardial Ischaemia National Audit Project; ^g^NCRAS, National Cancer Registration and Analysis Service.

VICORI includes over 6.2 million cancer diagnoses in 5.7 million patients, recorded between 1995 and 2018, and 3.8 million cardiac admissions and procedures, recorded between 1999 and 2018 in five of the six national cardiac audits (data from NCHDA is due to be included in 2021). Of these, 390 000 patients feature in both the cancer registry and one or more of the cardiac audits. Over 160 000 patients have both a cancer diagnosis and a hospital admission for acute coronary syndrome, as captured by MINAP. There are 61 000 patients with both cancer and heart failure and 85 000 with cancer and an implanted cardiac device. The overlap between the cancer registry and the available NICOR audits is shown in further detail in Figure 3. The bars show the distinct intersections between NCRAS and the different cardiac audits. The most prevalent linkages are between NCRAS and just one of the NICOR audits, for example, approximately 80 000 patients feature in both NCRAS and MINAP but in no other cardiovascular audit, whereas 52 000 patients feature in NCRAS, MINAP and NAPCI (and no other audit).

### Linkage and data quality

The linkages are detailed in Figure 1. Through deterministic linkage processes, patients in each NICOR audit are linked with: (i) other NICOR audits; (ii) the cancer registry (NCRAS); (iii) hospital admission records (HES); and (iv) death certification records (ONS). The VICORI resource additionally makes use of established linkages between the national cancer registration dataset, treatment datasets (SACT and RTDS), HES, and ONS death information. Linkage between the NICOR audits and NCRAS uses the 10-digit numerical NHS identifier (NHS number), which uniquely identifies patients in the UK. NICOR datasets are subject to a rigorous and fully documented cleaning pipeline, to ensure data quality and harmonization of data for use in analytical projects. Quality assurance of cancer registration data has already been implemented.^7^

### Anonymization and confidentiality

NICOR pseudonymizes NHS numbers using an SHA-256 hashing function, together with randomly derived cryptographic salt. Encrypted data from the six NICOR audits are transferred securely to PHE, which uses the same hashing algorithm (and salt) to pseudonymize NHS numbers for their cancer records. Linkage of the two data sources takes place within secure PHE working environments using the pseudonymized NHS number. The data are stored on a secure Oracle database (the Cancer Analysis System). PHE has permission to re-identify any cancer cases it holds data on, but non-cancer cases remain anonymous. The VICORI research team only have access to pseudonymized data.

Personal identifiers including names and addresses have been removed from the final dataset to protect privacy and confidentiality. Patient information which could facilitate re-identification, such as rare cancers (male breast cancer), or cases where sex is incompatible with tumour site, is not available in the final (analysis) dataset.

### Information governance and ethical permissions

PHE has been granted permission to collect information on cancer patients for health improvement and service provision without the need to seek consent, by Section 251 of the NHS Act 2006. Likewise, NICOR has corresponding approval for the collection of cardiovascular audit data. This is reviewed annually by the Confidentiality Advisory Group of the Health Research Authority.^22^ Approval for the VICORI linkage has been obtained from the Health Quality Improvement Partnership (HQIP) as data controller for the NICOR audits, PHE as data controller for NCRAS, and NHS Digital as data controller for HES and ONS, and is subject to data sharing agreements. Ethical approval for the VICORI programme has been obtained from the North East—Newcastle & North Tyneside 2 Research Ethics Committee (reference number 237503).

Applications to access VICORI data are made through application to the VICORI Project Review Panel (see Data resource access below) and then through formal application to the Office for Data Release.^23^ There are no restrictions on who can apply. However, applicants are required to demonstrate that they comply with UK data protection laws. This includes the Common Law Duty of Confidentiality,^24^ the General Data Protection Regulation (EU) 2016/679,^25^ and the seven Caldicott Principles.^26^ Applicants are required to abide by the terms of a data-sharing agreement, which includes obligations on data destruction following study completion. Applicants conducting research under the auspices of the VICORI collaborative ethical approval are also required to agree to abide by the terms of the protocol through a formal collaboration agreement.

### Data collection frequency and legacy

The cardio-oncology linkage will continue to be maintained by the study partners (NICOR, NCRAS and PHE). Follow-up in English EHR cohorts is initiated by capture within one or more of the audits or registries and will continue on a rolling basis. VICORI will receive annual periodic updates from HES, NICOR, NCRAS and ONS, thereby ensuring that VICORI will continue to be a leading, contemporary resource for cardio-oncology research beyond the 5-year programme grant. Furthermore, the linkages to ONS and HES allow long-term follow-up of patients who remain in England until their death, for all causes of death.

**Figure 4:**
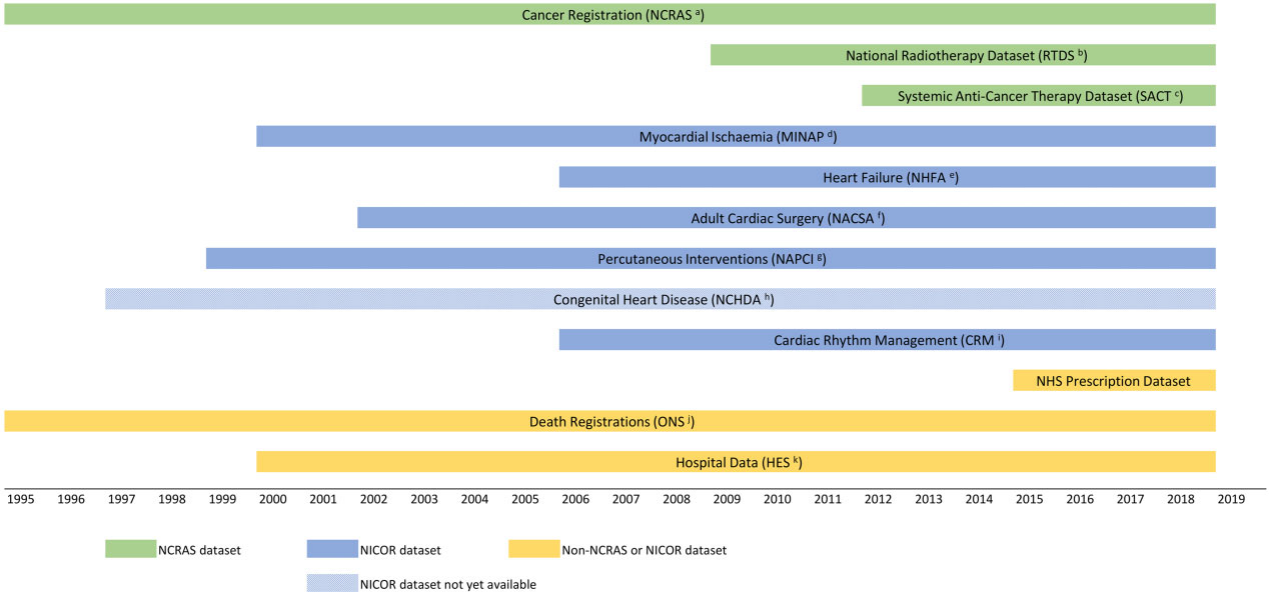
The follow-up duration available in each of the datasets in the Virtual Cardio-Oncology Research Initiative (VICORI) resource. ^a^NCRAS, National Cancer Registration and Analysis Service; ^b^RTDS, National Radiotherapy Dataset; ^c^SACT, Systemic Anti-Cancer Therapy Dataset; ^d^MINAP, Myocardial Ischaemia National Audit Project; ^e^NHFA, National Heart Failure Audit; ^f^NACSA, National Adult Cardiac Surgery Audit; ^g^NAPCI, National Audit of Percutaneous Coronary Interventions; ^h^NCHDA, National Congenital Heart Disease Audit; ^i^CRM, National Audit of Cardiac Rhythm Management; ^j^ONS, Office for National Statistics; ^k^HES, Hospital Episode Statistics.

The available follow-up time varies between datasets. The duration of each dataset is displayed in Figure 4. All datasets are available up to 2018–19, and all but RTDS, SACT and the prescription datasets have more than 10 years of data available.

## Data resource use

The VICORI programme is being used to address a wide range of research questions. It has four core work packages, each with a clinical lead. It will contribute new knowledge in the following four main areas:

- management of cardiac diseases, and outcomes after diagnosis of cardiac diseases, in patients with a previous cancer diagnosis. VICORI allows detailed investigation of cardiac management from the NICOR clinical audits. Example research question: how does the procedural management of patients with cancer who are hospitalized with MI differ from matched patients without a cancer diagnosis?
- risk of cancer after cardiovascular treatments, interventions and surgery. Example research question: are specific cardiovascular procedures particularly high risk for subsequent cancer development (e.g. chronic total occlusion angioplasty)?
- risk of cardiac disease in patients treated for cancer. Example research question: do cancer treatments increase the risk of subsequent cardiac disease?
- management of cancer and outcomes in cancer patients with pre-existing cardiac disease. Using linked NCRAS and HES data, the VICORI collaborative has demonstrated how CVD comorbidity plays a role in surgical resection rates in non-small-cell lung cancer patients and the potential undertreatment of CVD comorbid patients.^27^ Example research question: does previous valve surgery reduce adoption of a curative (surgical, chemotherapy, radiotherapy or targeted therapy) strategy for and timeliness of cancer treatment?

Furthermore, cross-cutting research is being undertaken to better understand the ascertainment of MI cases in the MINAP, HES and NAPCI datasets. However, this does not limit the use of VICORI data to solely those projects. Further avenues to understanding the various relationships and causal pathways between cardiac disease and cancer include investigating common risk factors, the impact of care delivery on CVD and cancer diagnoses and treatment, and evaluation of competing mortality risks in risk assessment.

VICORI data may also be used in the conduct of large-scale randomized controlled trials (RCTs) in cardio-oncology. This could include case ascertainment, recruitment, simplified protocols, easier outcome ascertainment and unrestricted long-term follow-up, and hence provide considerable cost savings.

## Strengths and weaknesses

One major strength of the data resource is the identification of a large cardio-oncological population. In contrast to investigator-led bespoke cohorts that focus on a narrow range of diseases, VICORI has the cohort size and clinical phenotyping to allow detailed investigations on a broad range of diseases. This is essential as causes, management and prognosis vary across different disease phenotypes. VICORI’s longitudinal, quality-assured data also allow temporal resolution, making it possible to distinguish whether an event was the index manifestation of cancer, for example, or if a previous diagnosis had been made. Finally, VICORI will be continually updated with new data on cancer and cardiac diagnoses, treatment and outcomes, thereby increasing the size of the cohort.

Some important limitations of the VICORI data have been identified. A key limitation of EHR, in general, is suboptimal data quality. Data quality in VICORI may be affected by missing and conflicting data. Some information, such as ethnicity, is less complete in some datasets (for example 21% of patients in NAPCI have missing ethnicity) but this is improved by linkages that allows information to be identified from other datasets. However, conflicting data may arise when data sources are combined. For example, MI hospital admissions may be recorded in MINAP, NAPCI and HES, which may differ in their timing accuracy and level of diagnostic detail. Linkage quality is another potential weakness. Linkage processes can introduce error through both false positives and false negatives,^28^^,^^29^ and affect the results of analyses.^30^ However, data entry into VICORI has been conducted by skilled cancer and CVD registration officers, and linkage, based on unique NHS numbers, is conducted by trained data developers. Finally, the data sources vary in their onset and duration (Figure 4). Whereas data are available in all datasets from 2012 onwards, the earliest dataset available for analysis in VICORI is the National Cancer Registration Dataset, which is available from 1995. This variation in time scales poses challenges in analyses and notably limits the assessment of detailed cancer treatment, as RTDS data are available from 2009 and SACT data from 2012.

## Data resource access

VICORI welcomes collaboration. For external researchers wishing to access data, full details of the application process, including data dictionaries, are available on the VICORI website [vicori.le.ac.uk]. Researchers can apply for access to linked VICORI data by completing an online form or contacting [vicori@le.ac.uk] in the first instance. An application for data access is subject to approval of a project proposal, analysis plan and data request by the VICORI Project Review Panel. The panel judge whether the proposal is: (i) achievable (nature and quality of the underlying data, the likely power of the study etc.) and deliverable; (ii) sufficiently distinct from other proposals and within remit; (iii) scientifically and methodologically rigorous; and (iv) has a purpose clinically relevant to cardio-oncology. If approved, a formal application is made to the Office for Data Release at PHE^23^ who, working with VICORI analysts, prepare a ‘research-ready’ bespoke dataset for the project and ensure strict data governance standards are met. To access the data, applicants must have a justified purpose for the data release, with an appropriate legal basis and safeguards in place to protect the data.

### Future data resource development

The VICORI data resources will grow in several key areas. The cohort will increase as more individuals are diagnosed and treated for cardiac diseases and cancer. Additional resources will be incorporated to provide greater depth to the data and the potential to investigate new research priorities. Potential extensions include linkage to the UK Renal Registry [https://renal.org/about-us/who-we-are/uk-renal-registry].

More broadly, VICORI points the way to a national NHS longitudinal data spine, with linkage by design between cardiology and cancer registry and other key data sources, e.g. primary care, social care and quality of life datasets.

## Funding

This manuscript was generated by the VICORI collaborative, which has been supported by grants from the British Heart Foundation (SP/16/5/32415) and Cancer Research UK (C53325/A21134).
